# Multilevel Analysis of Factors Associated with Wasting and Underweight among Children Under-Five Years in Nigeria

**DOI:** 10.3390/nu9010044

**Published:** 2017-01-08

**Authors:** Blessing J. Akombi, Kingsley E. Agho, Dafna Merom, John J. Hall, Andre M. Renzaho

**Affiliations:** 1 School of Science and Health, Western Sydney University, Locked Bag 1797, Penrith NSW 2571, Australia; K.Agho@westernsydney.edu.au (K.E.A.); D.Merom@westernsydney.edu.au (D.M.); 2 School of Medicine and Public Health, Faculty of Health, University of Newcastle, Callaghan NSW 2308, Australia; John.Hall@newcastle.edu.au; 3 School of Social Sciences and Psychology, Western Sydney University, Locked Bag 1797, Penrith NSW 2751, Australia; Andre.Renzaho@westernsydney.edu.au

**Keywords:** wasting, underweight, Nigeria, public health, malnutrition, multilevel analysis

## Abstract

Wasting and underweight reflect poor nutrition, which in children leads to retarded growth. The aim of this study is to determine the factors associated with wasting and underweight among children aged 0–59 months in Nigeria. A sample of 24,529 children aged 0–59 months from the 2013 Nigeria Demographic and Health Survey (NDHS) was used. Multilevel logistic regression analysis that adjusted for cluster and survey weights was used to identify significant factors associated with wasting/severe wasting and underweight/severe underweight. The prevalence of wasting was 18% (95% Confidence Interval (CI): 17.1, 19.7) and severe wasting 9% (95% CI: 7.9, 9.8). The prevalence of underweight was 29% (95% CI: 27.1, 30.5) and severe underweight 12% (95% CI: 10.6, 12.9). Multivariable analysis revealed that the most consistent factors associated with wasting/severe wasting and underweight/severe underweight are: geopolitical zone (North East, North West and North Central), perceived birth size (small and average), sex of child (male), place/mode of delivery (home delivery and non-caesarean) and a contraction of fever in the two weeks prior to the survey. In order to meet the WHO’s global nutrition target for 2025, interventions aimed at improving maternal health and access to health care services for children especially in the northern geopolitical zones of Nigeria are urgently needed.

## 1. Introduction

Malnutrition is a major public health problem faced by children under five years as it inhibits their cognitive and physical development as well as contributes to child morbidity and mortality [1]. Malnutrition is linked to poverty, low levels of education, poor access to health services and presence of infections. Protein-energy malnutrition (PEM) is the most common form of malnutrition and results from deficiencies in energy and protein intake. Stunting, wasting and underweight are expressions of PEM. These malnutrition indicators are caused by an extremely low energy and protein intake, nutrient losses due to infection, or a combination of both low energy/protein intake and high nutrient loss by the mother during pregnancy or by the child after birth [2].

The global prevalence for wasting and underweight decreased from 9% and 25% in 1990 to 8% and 14% in 2015, respectively [3]. Regionally, Africa and South Asia reported the highest rate of child malnutrition in the world accounting for about one third of all undernourished children globally. In Africa, 9.4% of children under-five years were wasted while 23.5% were underweight [3]. However, despite the global decrease, wasting and underweight in Nigeria have been on the rise in the past 10 years, with wasting increasing from 11% in 2003 to 18% in 2013 and underweight from 24% in 2003 to 29% in 2013 [4], as opposed to stunting, which, though a malnutrition indicator, has reported a decrease in Nigeria from 42% in 2003 to 37% in 2013 and globally by 37% between 1990 and 2015 [3]. This increase in wasting and underweight indicates a worsening in nutritional deficiency among children under-five years in the country, and thus necessitating the conduct of this study.

The factors associated with wasting and underweight are complex ranging from community-, household-, environmental-, socioeconomic and cultural influences as well as child feeding practises and presence of infections. Three cross-sectional studies conducted on 208 hospitalised children in south west Nigeria [5], 366 preschool children in northern Nigeria [6] and 119 under-five aged children in north western Nigeria [7] identified factors such as presence of infections, non-exclusive breastfeeding and low maternal education, diarrhoeal episode, father’s education and family size (>6) as strong determinants of wasting and underweight. However, these small scale studies were limited in scope as data used were not nationally representative. Hence, findings from such studies could not be generalised to the entire Nigerian population. Addressing wasting and underweight at early stages of child’s growth is of critical importance due to the heightened risk of morbidity and mortality among children with suboptimal energy availability.

This study utilised data from the 2013 National Demographic and Health Survey (NDHS) to determine the common predictors for wasting/severe wasting and underweight/severe underweight among Nigerian children aged 0–59 months and to describe the distribution of wasting and underweight by severity status across critical period of child growth. Thus, providing evidence on which interventions and policy actions can be formulated and implemented so Nigeria can achieve the World Health Assembly’s (WHA) Global Nutrition Target of reducing and maintaining childhood wasting to less than 5% and achieving a 30% reduction in low birth weight by 2025 [8].

## 2. Ethics

As this study was based on an analysis of existing survey datasets in the public domain that are freely available online with all identifier information removed, no ethics approvals were required. The first author obtained authorization for the download and usage of the NDHS dataset from MEASURE DHS/ICF International, Rockville, MD, USA.

## 3. Method

### 3.1. Data Sources

This study analysed data obtained from the 2013 NDHS. The survey was conducted by the National Population Commission (NPC) in collaboration with ICF Macro, Calverton, MD, USA [4].

In total, 40,680 households were selected for the survey with 39,902 women aged 15 to 49 years identified as eligible for individual interviews. Of which, 98% were successfully interviewed. A women’s questionnaire was used in recording the responses from all the women who participated in the survey.

In total, 30,050 children under the age of five were eligible for anthropometric measurements in all of the selected households. An overall 96% response rate was achieved with respect to height and weight measurements. Of the measurements carried out on the children, 88% were valid. This study focuses on the 24,529 children with valid and complete information on date of birth, height (cm) and weight (kg) [4].

Measurements were made using lightweight SECA scales (with digital screens) designed and manufactured under the authority of the United Nations Children’s Fund (UNICEF). The measuring boards employed for the measurement of height were specially made for use in survey settings. For children under 2 years, recumbent length was recorded while standing height was measured for older children.

### 3.2. Dependent Variables

#### 3.2.1. Wasting and Severe Wasting (Weight-for-Height)

The weight-for-height index measures body mass in relation to height and reflects current nutritional status. The index is calculated using growth standards published by the World Health Organization (WHO) in 2006. These growth standards were generated through data collected in the WHO Multicentre Growth Reference Study [9] and expressed in standard deviation units from the Multicentre Growth Reference Study median. Children with weight-for-height *Z*-scores below minus two standard deviations (−2 SD) from the median of the WHO reference population are considered wasted or acutely malnourished while children with *Z*-scores below minus three standard deviations (−3 SD) from the median of the WHO reference population are considered severely wasted.

#### 3.2.2. Underweight and Severe Underweight (Weight-for-Age)

Weight-for-age is a composite index of height-for-age and weight-for-height. It takes into account both acute malnutrition (wasting) and chronic malnutrition (stunting), but it does not distinguish between the two. Children whose weight-for-age is below minus two standard deviations (−2 SD) from the WHO Multicentre Growth Reference Study median [9] are classified as underweight. Children whose weight-for-age is below minus three standard deviations (−3 SD) from the reference median are considered severely underweight.

### 3.3. Independent Variables

**Figure 1:**
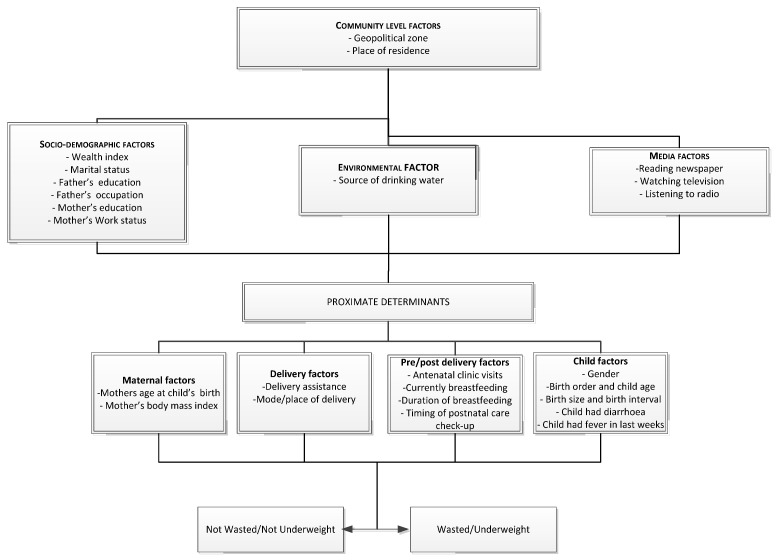
Conceptual framework for analysing factors associated with wasting/underweight and severe wasting/severe underweight in under-five aged children in Nigeria.

The potential risk factors were classified into five categories: Community level factors, socio-demographic factors, environmental factors, media factors and proximate determinants (Figure 1).

#### Adopted from UNICEF Conceptual Framework (2013)

Community level factors included geopolitical zone and type of residence (urban or rural). Geopolitical zones were defined based on ethnic homogeneity among states with similar cultures, history and close territories as well as political, administrative and commercial cities in Nigeria. The socio-demographic, environmental and media factors are as represented in Figure 1. Household wealth index serves as an indicator consistent with expenditure and income measures. It was represented as a score of household assets via the principle components analysis method (PCA) [10]. Once this index was computed, scores were assigned to each de jure household member, ranking each person in the population by his or her score. The index was categorized into five national-level wealth quintiles: poorest, poor, middle, rich and richest. The bottom 40% of the households was referred to as the poorest and poor households, the next 20% as the middle-class households, and the top 40% as rich and richest households. Environmental factor was source of drinking water which was categorized into improved and unimproved according to WHO/UNICEF guidelines [11]. The proximate determinants were subdivided into maternal factors, delivery factors, pre/post-delivery factors and child factors (Figure 1). A combination of place of delivery and mode of delivery was further subdivided into three categories: home delivery, delivery at health facility with non-caesarean and delivery at health facility with caesarean.

### 3.4. Statistical Analysis

The indicator for wasting and underweight was expressed as a dependent dichotomous variable as follows:

*Undernutrition*; Category 0 (not wasted/not underweight (>−2 SD)) and category 1 (wasted/underweight (>−3 SD)).
*Severe undernutrition*; Category 0 (not severely wasted/not severely underweight (>−2 SD)) and category 1 (severely wasted/severely underweight (>−3 SD)).

These were examined against the set of independent variables in order to determine the factors associated with wasting/underweight and severe wasting/severe underweight in children under-five years.

**Figure 2:**
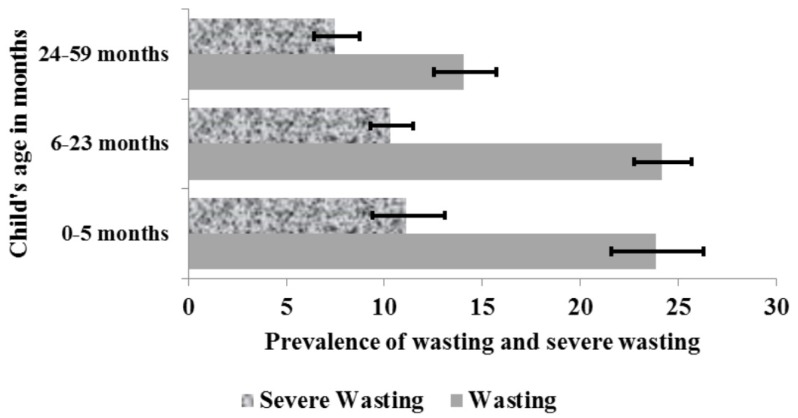
Prevalence of wasting and severe wasting by child’s age in months.

**Figure 3:**
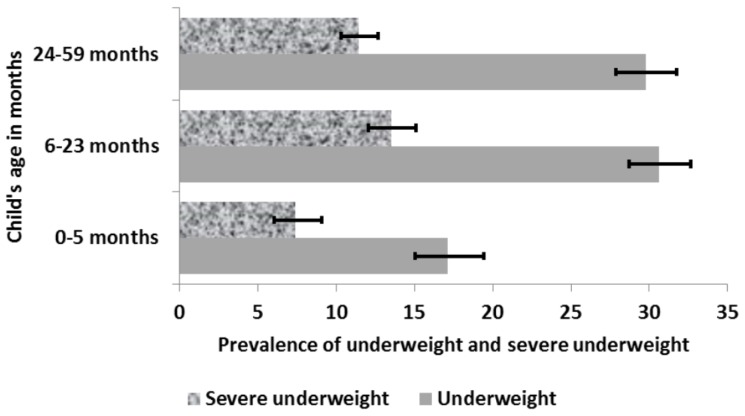
Prevalence of underweight and severe underweight by child’s age in months.

Analysis was performed using Stata version 14.0 (StataCorp, College Station, TX, USA). The confidence intervals (CIs) around prevalence estimates of children aged 0–6 months, 6–23 months and 24–59 months was estimated using the Taylor series linearization method as reported in Figure 2 and Figure 3. These age groups were chosen because exclusive breastfeeding in the first six month of life, appropriate complementary feeding practices among children aged 6–23 months, and adequate psychosocial stimulation for children aged 24–59 months are important factors in reducing malnutrition.

Logistic regression generalized linear latent and mixed models (GLLAM) with the logit link and binomial family [12] that adjusted for cluster and survey weights were used to identify the factors associated with wasting/severe wasting and underweight/severe underweight amongst children aged 0–59 months.

Multivariable analysis was conducted using a five-stage conceptual modelling technique adopted from UNICEF [13] (Figure 1). The first stage involved entering community level factors into the baseline model to determine their association with the outcome variables. A stepwise backward elimination was performed and factors significantly associated with the study outcomes were retained. In the second modelling stage, socio-demographic factors were added to the significant factors from the first model and the backward elimination procedure was repeated. This protocol was followed for the inclusion of environmental factors, media factors and proximate determinants in the third, fourth, and fifth modelling stages respectively. In each stage, the factors with *p*-values < 0.05 were retained. To avoid any statistical bias, we confirmed our results by: (1) performing a backward elimination process on potential risk factors with a *p*-value < 0.20 obtained in the univariable analysis; (2) testing the backward elimination method by including all of the variables (all potential risk factors); and (3) testing and reporting collinearity. In order to determine the adjusted risk of the independent variables, the odds ratios with 95% CI were calculated and those with *p* < 0.05 were retained in the final model.

## 4. Results

The prevalence of wasting and severe wasting among children aged 0–59 months was 18% (95% CI: 17.1, 19.7) and 9% (95% CI: 7.9, 9.8), respectively. An analysis of the distribution of wasting by child’s age in months showed that children aged 0–23 months were more wasted and severely wasted than children aged 24–59 months (Figure 2).

Underweight and severe underweight among children aged 0–59 months was 29% (95% CI: 27.1, 30.5) and 12% (95% CI: 10.6, 12.9), respectively. Underweight and severe underweight was less predominant in children aged 0–5 months and highest among children aged 6–23 months as shown in Figure 3.

A total sample of 24,529 children aged 0–59 months was included in the study. Table 1 below shows the characteristics of the sample.

## 5. Multivariate Analysis

Table 2 summarises the unadjusted and adjusted odds ratios (OR) for the association between the independent variables and wasting (moderate and severe), while Table 3 shows the corresponding OR for underweight and severe underweight, respectively.

### 5.1. Factors Associated with Wasting and Severe Wasting

Children residing in rural areas and in the North West geopolitical zone were significantly more predisposed to wasting and severe wasting than those in urban areas and other geopolitical zones. Children of uneducated parents and living in households that do not watch television had significantly higher odds of being wasted and severely wasted compared with those of educated parent and exposed to the media. Children who were delivered at home and children who were perceived to be small by their mothers at birth were more likely to be wasted and severely wasted than those delivered at a health facility and perceived to have been large. Male children and mothers with BMI less than 18.5 kg/m^2^ were significantly more susceptible to wasting and severe wasting than their female counterparts and mothers with BMI greater than 18.5 kg/m^2^. Children who were delivered with no assistance from health professionals and children who had fever in the two weeks preceding the survey were more likely to be wasted compared with children who had assisted delivery and who did not have fever. Child’s age was also significantly associated with wasting and severe wasting.

### 5.2. Factors Associated with Underweight and Severe Underweight

Children residing in the North West geopolitical zone, and born to uneducated parents were significantly more likely to be underweight and severely underweight compared with those who were born to educated parents and reside in other geopolitical zones. Children who were delivered at home, and whose mothers had BMI less than 18.5 kg/m^2^ were significantly more likely to be underweight and severely underweight compared with those delivered at a health facility and whose mothers had BMI greater than 18.5 kg/m^2^. Children who had a prolonged period of breastfeeding (>12 months), and children whose mothers reported a preceding birth interval of less than 24 months were more likely to be underweight and severely underweight compared with those who were breastfed for less than 12 months and had more than 24 months birth interval. Male children were more likely to be underweight and severely underweight compared with their female counterparts. Children who were perceived by their mothers to have been small at birth, and children who were not being breastfed were more likely to be underweight and severely underweight than those that were perceived to have been large and were being breastfed. Children who had diarrhoea and fever in the two weeks prior to the survey were significantly more likely to be underweight and severely underweight compared with those who had neither diarrhoea nor fever. Child’s age was significantly associated with severe underweight.

## 6. Discussion

Our analysis reported that children aged 0–5 months and 6–23 months are the most affected by wasting and severe wasting (Figure 2) while children aged 6–23 months and 24–59 months are the most affected by underweight and severe underweight (Figure 3). Inadequate nutrition in the first two years of life leads to acute weight loss and prevents the child from developing at a rate where its body weight is commensurate to its height. The mother’s nutritional status is very important to the proper development of the child in utero and continues to be for at least the first six months of post-natal life when the child is totally dependent on the mother for all its nutrient supply. Failure of the mother to exclusively breastfeed the child in the first six months may lead to growth deficit [14]. After six months, a child requires adequate complementary foods for optimal growth [15,16]. The period of transition from exclusively breastfeeding (0–6 months) to the introduction of complementary foods (6–23 months) is a very critical period where the child is most vulnerable to malnutrition. Prolonged breastfeeding without the timely introduction of supplementary foods that is of good quality, quantity and at the right frequency to cater for the nutritional needs of the growing child while maintaining breastfeeding may result in undernutrition and frequent illness [17]. This finding is consistent with WHO recommendation that infants should start receiving adequate complementary foods at 6 months of age in addition to breast milk to avoid being malnourished [17]. Furthermore, children aged 24–59 months require more energy (calories) and nutrients for proper growth and development. As the child grows, its energy needs increases and so should its energy (calories) intake in order to maintain the appropriate weight for its age. It is therefore crucial they obtain their daily energy from a varied, healthy and balanced diet. Inability to meet the growing energy and nutrient needs of the child results in the child being underweight.

In this study, children who resided in the North East, North West and North Central geopolitical zones of Nigeria had a significantly higher risk of being wasted and underweight. This could either be due to political unrest in the region or the neglect of agriculture as well as the effect of cultural preferences on food choice where certain types of food are not given to children even though the food are nutritious, but instead the children are fed a monotonous rice-based native meal with low nutrient all year round [18]. This has led to the recent concerns of the Nigerian government with the level of malnutrition in the Northern region of the country [19]. A similar cross-sectional study carried out in the Democratic Republic of Congo (DRC) revealed that malnutrition rates remain very high in provinces that rely on the mining industry (Katanga, the two Kasai and the Orientale) as the younger generation has left the agricultural sector to work in the mining industries. These rates where comparable to the level seen in the Eastern provinces under war as people do not cultivate due to the violence [20].

The mother’s perception of the birth size of their child was significantly associated with the child’s nutritional status. Children who were perceived to be small at birth were more susceptible to wasting as well as being underweight compared with those perceived to be large; this is consistent with results of previous studies in Ethiopia [21], Brazil [22] and Pakistan [23] that reported birth size as a valid indicator of subsequent growth. However, caution should be taken in interpreting this result, as the rationale used by the mothers in estimating the size of their babies is unclear. Reduced birth size maybe a result of poor maternal nutrition during pregnancy when the child is totally dependent on the mother for its nutrition in utero via the placenta, thus any nutrition deprivation from the mother will affect the growth and proper development of the foetus [4]. This finding thus highlights the importance of women’s health and prenatal care for giving their offspring a better chance in life.

In this study, male children had a significantly higher risk of being wasted and underweight than their female counterparts. Male children tend to engage in higher intensity physical activity thereby using up large amounts of energy that was meant for proper growth and development. Meanwhile, female children are culturally expected to perform lower intensity physical activity which includes staying at home with their mothers near food preparation thereby conserving and channelling more energy to growth and development. This finding is consistent with results from other cross-sectional studies carried out in Ethiopia [21] and South Africa [24] which also found that males’ were more likely to be undersized and underweight than females. However, a biological reason for this is still unknown.

In this study, place of delivery significantly increased a child’s vulnerability to wasting and underweight. Children delivered at home tend to have poorer nutritional status than children delivered at a health facility. Studies have shown a strong association between institutional delivery and mother’s education, which in turn affects child health [25,26]. Home delivery is mostly practised by women of lower educational status [26]; these women tend to lack the necessary knowledge needed to make informed decisions concerning the health of their child. Women who deliver at home also miss out on the valuable post-natal counselling provided at the health facilities, which may help in improving the nutritional status of both mother and child.

This study also revealed that children who suffered a contraction of fever or diarrhoea in the two weeks preceding the survey tend to be more nutritionally deprived than children who did not. The occurrence of fever or diarrhoea and malnutrition are interrelated; fever and diarrhoea tend to reduce appetite and interfere with the digestion and absorption of food consumed which in turn exacerbates malnutrition thus directing essential nutrients away from growth towards immune response thereby leading to growth failure [27]. In a recent cross-sectional study conducted in Ethiopia, it was discovered that the children who had fever two weeks prior to the survey showed poorer nutritional status [21]. Another study conducted in South Ethiopia reported that the presence of diarrhoea in under-five year old children two weeks prior to the survey was significantly associated with malnutrition [28].

Children whose mothers had a BMI less than 18.5 kg/m^2^ were significantly more likely to be wasted and underweight than those whose mothers had a BMI of 25 kg/m^2^ or higher. Mother’s BMI is an important determinant of malnutrition in children, therefore supplementary food for the mothers in the prenatal and postnatal period is recommended in order to improve child growth. A similar cross-sectional study conducted in Ethiopia reported that the mother’s BMI, which is an indicator of the mother’s nutritional status, was significantly associated with wasting in their offspring [21].

In this study, children whose parents resided in rural areas were more undernourished than those residing in the urban areas. Health facilities in rural areas are often ill-equipped for delivering the required primary health care services [20]. Rural areas also lack access to safe water supply, proper housing and adequate sanitation, which are preconditions for adequate nutrition and directly affect health. This inequality results in a greater susceptibility to infections and slow recovery from illness thereby adversely affecting growth. This finding is consistent with results from a cross-sectional study carried out in the DRC, which also found that the rate of malnutrition was significantly higher in rural areas compared to urban areas [20].

Children born to uneducated parents tend to be at a higher risk of malnutrition than children born to educated parents. This result supports the potential link of maternal education to child health. A higher maternal education translates into greater health care utilization, including formal prenatal and postnatal visits. It exposes mothers to a better understanding of diseases and adoption of modern medical practices. Higher maternal education leads to greater female autonomy, which in turn influences health-related decisions and the allocation of resources for food within the household [26]. Education on the nutritional value of foods and the best way of food preparation add to improving the nutritional status of the child. In a cross-sectional study conducted in Kenya, higher maternal education was reported to be associated with maternal employment and higher household income [29], which in turn improves the child’s access to good quality food. Similarly, father’s education also translates to a higher household income and food security. Previous cross-sectional studies conducted in Zambia [30], Iran [31] and Nepal [32] on the relationship between wealth index and malnutrition reported that children from poor households were more likely to be undernourished than those from rich households. This may be attributed to the fact that with less income to spend on proper nutrition, children from underprivileged households are more susceptible to growth failure due to insufficient food intake.

Children from households exposed to the media (television) are less prone to wasting and severe wasting as their parents are socially more advanced and tend to be more exposed to important information about proper nutrition and child feeding practices. This finding is similar to that of a cross-sectional study conducted in Bangladesh which highlighted a positive relationship between the media and wasting [33].

Our study had several strengths. Firstly, the study was population-based with a large sample size that yielded a 96% and 98% response rate for children and women respectively. Secondly, the study used the 2013 NDHS dataset, which is the most recent nationally recognised data available in Nigeria thereby giving relevance to the study. Thirdly, appropriate statistical adjustments were applied to the 2013 NDHS dataset and the most vulnerable subpopulation affected by wasting/severe wasting or underweight/severe underweight was identified. However, the study was limited in a number of ways. Firstly, we were unable to establish a causal relationship between the observed risk factors and the dependent variables due to the cross-sectional nature of the study design. Secondly, despite the use of a comprehensive set of variables in our analysis, the effect of residual confounding as a result of unmeasured co-variates could not be ruled out; this include direct measures of child’s diet and feeding pattern as well as energy expenditure through physical activity to identify possible casual paths.

### Policy Implications

Intervention strategies geared towards improving mother’s knowledge about exclusive breastfeeding and adequate complimentary feeding practices should be implemented and should target mothers from poor socio-economic group. The Nigerian government should also focus on provision of accessible health care services to all mothers especially those from the northern geopolitical region of the country.

Findings from this study will enable policy makers and public health researchers to develop effective nutrition interventions targeting the most vulnerable subpopulation that could be translated into policy actions to reduce the double burden of malnutrition in Nigeria.

## 7. Conclusions

Considering the findings in this study, it is critical that community-based interventions need to be formulated and implemented in order to improve child health. At the individual level, interventions should focus on educating mothers on the basics of proper nutrition and the need to utilize available health services. At the community level, healthcare systems that facilitate public health interventions such as maternal-and-child health programs need to be made accessible to women in rural areas. These interventions will improve the nutritional status of children under-five years in Nigeria, thereby setting the country on the path to achieving the WHO global nutrition target by 2025.

## Figures and Tables

**Table 1:** Characteristics of independent variables.

Characteristics	*n*	%
***Community Level Factors***		
**Type of residence**		
Urban	9067	37.0
Rural	15,465	63.0
**Geopolitical Zones**		
North Central	3562	14.5
North East	4086	16.7
North West	8506	34.7
South East	2284	9.3
South West	2372	9.7
South South	3722	15.2
***Socio-demographic factors***		
**Wealth Index**		
Poorest	5378	21.9
Poor	5383	21.9
Middle	4711	19.2
Rich	4598	18.7
Richest	4462	18.2
**Mother’s working status**		
Non-working	16,151	97.1
Working (past 12 months)	485	2.9
**Maternal education**		
No education	11,378	46.4
Primary	4933	20.1
Secondary and above	8221	33.5
**Father’s occupation**		
Non agriculture	20,237	82.5
Agriculture	1024	4.2
Not working	3271	13.3
**Father’s education**		
No education	8870	37.0
Primary	4640	19.4
Secondary and above	10,447	43.6
**Marital status**		
Currently married	23,592	97.6
Formerly married (Divorce/Separated/Widow)	579	2.4
**Mother’s literacy**		
Can’t read at all	14,029	57.5
Can read	10,386	42.5
***Environmental factor***		
**Source of drinking water**		
Protected	13,878	56.6
Unprotected	10,653	43.4
***Media factors***		
**Reading newspaper**		
Yes	3589	14.7
No	20,793	85.3
**Listening to radio**		
Yes	15,135	61.9
No	9314	38.1
**Watching TV**		
Yes	11,690	47.9
No	12,732	52.1
***Proximate determinants***		
***Maternal factors***		
**Mother’s age**		
15–24 years	5780	23.6
25–34 years	12,424	50.6
35–49 years	6328	25.8
**Mother’s age at birth**		
<20 years	3325	13.6
20–29 years	12,878	52.5
30–39 years	7161	29.2
40 and above	1168	4.8
***Delivery factors***		
**Type of delivery assistance**		
Health professional	10,399	42.8
Traditional birth attendant	4938	20.3
Relatives and other untrained personnel	5856	24.1
No one	3113	12.8
**Place of delivery**		
Home	15,065	61.4
Health facility	9467	38.6
**Mode of delivery**		
Non-caesarean	23,734	97.8
Caesarean	523	2.2
**Combined Place and mode of delivery**		
Non-caesarean and Home delivery	15,065	62.1
Non-caesarean & Health facility	8669	35.7
Caesarean & Health facility	523	2.2
***Pre/post-delivery factors***		
**Antenatal clinic visits**		
None	5177	32.8
1–3	1954	12.4
4+	8674	54.9
**Timing of postnatal check-up**		
No postnatal check-up	19,243	78.4
0–2 days	3748	15.3
Delayed	1541	6.3
**Currently breastfeeding**		
Yes	13,950	56.9
No	10,582	43.1
**Duration of breastfeeding**		
up to 12 months	5376	22.3
>12 months	18,792	77.8
***Child factors***		
**Birth order**		
First-born	4641	19.0
2nd–4th	11,327	46.2
5 or more	8564	34.9
**Preceding birth interval**		
No previous birth	4641	19.0
<24 months	4326	17.7
>24 months	15,520	63.4
**Sex of child**		
Male	12,193	49.7
Female	12,339	50.3
**Perceived birth size**		
Small	3385	14.0
Average	10,052	41.5
Large	10,759	44.5
**Child’s age in months**		
0–5	2238	9.3
6–23	7876	32.8
24–59	13,915	57.9
**Child had diarrhoea recently**		
No	21,885	89.3
Yes	2556	10.4
**Child had fever in last two weeks**		
No	21,251	86.6
Yes	3153	12.9

**Table 2:** Unadjusted and adjusted odds ratios (OR) (95% CI) for wasted and severely wasted children aged 0–59 months.

Characteristics	Wasted Children 0–59 Months				Severely Wasted Children 0–59 Months			
	Unadjusted Odd Ratio (95% CI)	*p*	Adjusted Odd Ratio (95% CI)	*p*	Unadjusted Odd Ratio (95% CI)	*p*	Adjusted Odd Ratio (95% CI)	*p*
***Community Level Factors***								
**Type of residence**								
Urban	1.00		1.00		1.00		1.00	
Rural	1.05 (0.86, 1.28)	0.641	0.72 (0.59, 0.89)	0.001	1.07 (0.81, 1.41)	0.641	0.71 (0.55, 0.93)	0.013
**Geopolitical Zones**								
North Central	1.00		1.00		1.00		1.00	
North East	1.73 (1.38, 2.17)	<0.001	1.51 (1.19, 1.91)	0.001	2.07 (1.46, 2.94)	<0.001	1.87 (1.31, 2.66)	0.001
North West	2.59 (2.09, 3.22)	<0.001	2.42 (1.93, 3.03)	<0.001	3.60 (2.62, 4.95)	<0.001	3.17 (2.28, 4.40)	<0.001
South East	0.98 (0.77, 1.23)	0.837	0.81 (0.63, 1.05)	0.112	0.91 (0.63, 1.32)	0.613	0.69 (0.47, 1.04)	0.074
South West	0.92 (0.72, 1.16)	0.473	0.88 (0.69, 1.12)	0.285	0.82 (0.56, 1.21)	0.316	0.75 (0.51, 1.10)	0.143
South South	0.78 (0.62, 0.98)	0.031	0.67 (0.52, 0.85)	0.001	0.61 (0.42, 0.87)	0.007	0.49 (0.33, 0.71)	<0.001
***Socio-demographic factors***								
**Mother’s education**								
No education	1.00		1.00		1.00		1.00	
Primary	0.65 (0.57, 0.74)	<0.001	0.90 (0.78, 1.04)	0.160	0.53 (0.44, 0.63)	<0.001	0.81 (0.66, 0.98)	0.002
Secondary and above	0.54 (0.47, 0.62)	<0.001	0.79 (0.67, 0.94)	0.007	0.47 (0.38, 0.58)	<0.001	0.74 (0.58, 0.94)	0.014
**Father’s education**								
No education	1.00		1.00		1.00		1.00	
Primary	0.67 (0.58, 0.76)	<0.001	0.89 (0.77, 1.03)	0.120	0.57 (0.47, 0.70)	<0.001	0.84 (0.68, 1.03)	0.095
Secondary and above	0.57 (0.50, 0.65)	<0.001	0.77 (0.67, 0.88)	<0.001	0.47 (0.39, 0.58)	<0.001	0.65 (0.53, 0.79)	<0.001
**Watching TV**								
Yes	1.00		1.00		1.00		1.00	
No	1.28 (1.13, 1.46)	<0.001	0.78 (0.68, 0.88)	<0.001	1.30 (1.08, 1.57)	0.005	0.68 (0.56, 0.82)	<0.001
***Proximate determinants***								
***Maternal factors***								
**Mother’s BMI**								
<18.5	1.00		1.00		1.00			
18.5–25	0.66 (0.56, 0.78)	<0.001	0.76 (0.64, 0.90)	0.002	0.73 (0.59, 0.91)	0.005		
25+	0.48 (0.39, 0.59)	<0.001	0.68 (0.56, 0.83)	<0.001	0.56 (0.43, 0.74)	<0.001		
***Delivery factors***								
**Type of delivery assistance**								
No one	1.00		1.00		1.00			
Traditional birth attendant	1.85 (1.59, 2.15)	<0.001	1.39 (1.11, 1.73)	0.004	2.25 (1.79, 2.82)	<0.001		
Relatives or other	1.77 (1.53, 2.05)	<0.001	1.44 (1.14, 1.80)	0.002	2.08 (1.74, 2.49)	<0.001		
Health professional	1.84 (1.55, 2.19)	<0.001	1.11 (0.88, 1.41)	0.367	2.39 (1.88, 3.04)	<0.001		
**Combined Place/mode of delivery**								
Home delivery	1.00		1.00		1.00		1.00	
Health facility with non-caesarean	0.59 (0.52, 0.67)	<0.001	1.12 (0.92, 1.37)	0.254	0.47 (0.39, 0.57)	<0.001	0.79 (0.65, 0.96)	0.017
Health facility with caesarean	0.31 (0.20, 0.46)	<0.001	0.61 (0.39, 0.94)	0.025	0.24 (0.14, 0.43)	<0.001	0.44 (0.24, 0.79)	0.007
***Child factors***								
**Sex of child**								
Male	1.00		1.00		1.00		1.00	
Female	0.88 (0.81, 0.95)	0.001	0.83 (0.77, 0.89)	<0.001	0.85 (0.76, 0.94)	0.002	0.79 (0.71, 0.88)	<0.001
**Perceived birth size**								
Small	1.00		1.00		1.00		1.00	
Average	0.76 (0.66, 0.87)	<0.001	0.85 (0.74, 0.97)	0.017	0.72 (0.60, 0.85)	<0.001	0.81 (0.68, 0.96)	0.018
Large	0.60 (0.52, 0.69)	<0.001	0.66 (0.57, 0.76)	<0.001	0.57 (0.48, 0.67)	<0.001	0.64 (0.53, 0.77)	<0.001
**Child had fever in last two weeks**								
No	1.00		1.00		1.00			
Yes	1.07 (0.94, 1.21)	0.312	1.18 (1.06, 1.32)	0.003	0.81 (0.68, 0.98)	0.028		
**Child’s age in months**	0.98 (0.98, 0.98)	<0.001	0.98 (0.98, 0.98)	<0.001	0.99 (0.98, 0.99)	<0.001	0.98 (0.98, 0.99)	<0.001

Independent variables adjusted for are: Type of residence, geopolitical zones, wealth index, mother’s working status, maternal education, fathers occupation, father’s education, marital status, mother’s literacy, source of drinking water, reading newspaper, listening to radio, watching TV, mother’s age, mother’s age at birth, type of delivery assistance, combined place and mode of delivery, antenatal clinic visits, timing of postnatal check-up, currently breastfeeding, duration of breastfeeding, birth order, preceding birth interval, sex of child, perceived birth size, child’s age in months, child had diarrhoea recently, child had fever in last two weeks.

**Table 3:** Unadjusted and adjusted odds ratios (OR) (95% CI) for underweight and severely underweight children aged 0–59 months.

Characteristics	Underweight Children 0–59 Months				Severely Underweight Children 0–59 Months			
	Unadjusted Odd Ratio (OR) (95% CI)	*p*	Adjusted Odd Ratio (AOR) (95% CI)	*p*	Unadjusted Odd Ratio (OR) (95% CI)	*p*	Adjusted Odd Ratio (AOR) (95% CI)	*p*
***Community Level Factors***								
**Geopolitical Zones**								
North Central	1.00		1.00		1.00		1.00	
North East	1.97 (1.58, 2.45)	<0.001	1.44 (1.17, 1.78)	0.001	1.85 (1.34, 2.56)	<0.001	1.48 (1.06, 2.06)	0.021
North West	3.94 (3.20, 4.84)	<0.001	3.22 (2.58, 4.01)	<0.001	4.37 (3.19, 5.98)	<0.001	3.82 (2.72, 5.35)	<0.001
South East	0.52 (0.39, 0.67)	<0.001	0.47 (0.36, 0.59)	<0.001	0.38 (0.24, 0.58)	<0.001	0.32 (0.21, 0.49)	<0.001
South West	0.64 (0.51, 0.81)	<0.001	0.64 (0.51, 0.79)	<0.001	0.49 (0.32, 0.75)	0.001	0.48 (0.32, 0.73)	0.001
South South	0.73 (0.58, 0.93)	0.011	0.76 (0.60, 0.96)	0.022	0.53 (0.35, 0.80)	0.003	0.52 (0.34, 0.78)	0.002
***Socio-demographic factors***								
**Mother’s education**								
No education	1.00		1.00		1.00			
Primary	0.48 (0.43, 0.54)	<0.001	0.89 (0.79, 1.01)	0.062	0.49 (0.41, 0.58)	<0.001		
Secondary and higher	0.29 (0.26, 0.35)	<0.001	0.74 (0.62, 0.87)	<0.001	0.28 (0.21, 0.36)	<0.001		
**Father’s education**								
No Education	1.00		1.00		1.00		1.00	
Primary	0.56 (0.49, 0.63)	<0.001	0.96 (0.85, 1.08)	0.500	0.62 (0.52, 0.74)	<0.001	1.14 (0.95, 1.36)	0.153
Secondary and higher	0.38 (0.33, 0.43)	<0.001	0.78 (0.69, 0.88)	<0.001	0.38 (0.31, 0.46)	<0.001	0.85 (0.71, 1.03)	0.103
***Proximate determinants***								
***Maternal factors***								
**Mother’s BMI**								
<18.5	1.00		1.00		1.00		1.00	
18.5–25	0.56 (0.48, 0.66)	<0.001	0.67 (0.57, 0.78)	<0.001	0.64 (0.53, 0.78)	<0.001	0.78 (0.63, 0.95)	0.015
25+	0.30 (0.25, 0.37)	<0.001	0.52 (0.43, 0.63)	<0.001	0.35 (0.28, 0.46)	<0.001	0.66 (0.51, 0.84)	0.001
***Delivery factors***								
**Combined Place/mode of delivery**								
Home delivery	1.00		1.00		1.00		1.00	
Health facility with non-caesarean	0.38 (0.33, 0.43)	<0.001	0.85 (0.76, 0.95)	<0.001	0.34 (0.28, 0.41)	<0.001	0.69 (0.52, 0.91)	0.008
Health facility with caesarean	0.26 (0.18, 0.37)	<0.001	0.69 (0.48, 0.99)	0.016	0.27 (0.16, 0.47)	<0.001	0.67 (0.33, 1.36)	0.268
**Currently breastfeeding**								
No	1.00		1.00		1.00			
Yes	0.85 (0.79, 0.92)	<0.001	0.89 (0.81, 0.97)	0.007	0.84 (0.75, 0.95)	0.005		
**Duration of breastfeeding**								
up to 12 months	1.00		1.00		1.00		1.00	
>12 months	1.36 (1.24, 1.49)	<0.001	1.61 (1.44, 1.80)	<0.001	1.22 (1.07, 1.38)	0.002	1.91 (1.64, 2.23)	<0.001
***Child factors***								
**Preceding birth interval**								
No previous birth	1.00		1.00		1.00		1.00	
<24 months	1.32 (1.18, 1.48)	<0.001	1.11 (0.98, 1.26)	0.093	1.48 (1.23, 1.77)	<0.001	1.29 (1.06, 1.56)	0.010
>24 months	1.16 (1.05, 1.27)	0.002	0.97 (0.88, 1.07)	0.513	1.25 (1.09, 1.44)	0.002	1.04 (0.89, 1.19)	0.620
**Sex of child**								
Male	1.00		1.00		1.00		1.00	
Female	0.86 (0.80, 0.92)	<0.001	0.79 (0.74, 0.85)	<0.001	0.86 (0.78, 0.95)	0.002	0.79 (0.72, 0.88)	<0.001
**Perceived birth size**								
Small	1.00		1.00		1.00		1.00	
Average	0.72 (0.64, 0.82)	<0.001	0.87 (0.76, 0.99)	0.044	0.66 (0.56, 0.77)	<0.001	0.78 (0.65, 0.93)	0.005
Large	0.49 (0.43, 0.55)	<0.001	0.55 (0.48, 0.63)	<0.001	0.44 (0.37, 0.52)	<0.001	0.50 (0.41, 0.60)	<0.001
**Child had diarrhoea recently**								
No	1.00		1.00		1.00		1.00	
Yes	1.51 (1.35, 1.69)	<0.001	1.36 (1.21, 1.53)	<0.001	1.55 (1.34, 1.78)	<0.001	1.43 (1.24, 1.65)	<0.001
**Child had fever in last two weeks**								
No	1.00		1.00		1.00		1.00	
Yes	1.28 (1.15, 1.43)	<0.001	1.35 (1.21, 1.51)	<0.001	1.13 (0.96, 1.32)	0.137	1.22 (1.03, 1.46)	0.024
**Child’s age in months**	1.00 (1.00, 1.01)	0.002			0.99 (0.99, 0.99)	0.002	0.98 (0.98, 0.99)	<0.001

Independent variables adjusted for are: Type of residence, geopolitical zones, wealth index, mother’s working status, maternal education, fathers occupation, father’s education, marital status, mother’s literacy, source of drinking water, reading newspaper, listening to radio, watching TV, mother’s age, mother’s age at birth, type of delivery assistance, combined place and mode of delivery, antenatal clinic visits, timing of postnatal check-up, currently breastfeeding, duration of breastfeeding, birth order, preceding birth interval, sex of child, perceived birth size, child’s age in months, child had diarrhoea recently, child had fever in last two weeks.
